# Mutations in the dynein assembly factor PF22 (DNAAF3) cause primary ciliary dyskinesia with absent dynein arms

**DOI:** 10.1186/2046-2530-1-S1-P101

**Published:** 2012-11-16

**Authors:** M Schmidts, J Freshour, NT Loges, A Dritsoula, D Antony, RA Hirst, C O’Callaghan, H Blau, H Olbrich, T Yagi, H Mussaffi, EMK Chung, H Omran, DR Mitchell, H Mitchison

**Affiliations:** 1 Molecular Medicine Unit, UCL Institute of Child Health, UK; 2 Cell & Developmental Biology, SUNY Upstate Medical University, USA; 3 Department of Pediatrics and Adolescent Medicine, University Hospital Freiburg and University Hospital Muenster, Germany; 4 Department of Infection Immunity and Inflammation, University of Leicester,UK; 5 Pulmonary Unit, Schneider Children’s Medical Center of Israel, Israel; 6 Department of Cell Biology and Anatomy, Graduate School of Medicine, University of Tokyo, Japan; 7 General and Adolescent Paediatric Unit, UCL Institute of Child Health, UK

## 

The genetic disorder primary ciliary dyskinesia (PCD) arises from dysmotility of cilia in the respiratory tract, brain ventricles, oviduct and the embryonic node. Patients have chronic obstructive pulmonary disease, reduced fertility and situs abnormalities. PCD is genetically heterogeneous with 12 genes causing ~40% of all cases, two encoding proteins (KTU, LRRC50) involved in cytosolic axonemal dynein co-assembly. We have identified mutations in the *C19ORF51* gene located within a previously mapped PCD locus. *C19ORF51* encodes a protein orthologous to PF22, a Chlamydomonas protein involved in the cytoplasmic assembly of outer dynein arms preceeding their import into the axoneme. Chlamydomonas pf22 cells display a disturbance in their cytoplasm of dynein heavy chain stability and the co-assembly of heavy with intermediate chains, both essential for dynein arm assembly. PF22 appears to act downstream of KTU and LRRC50 in the dynein preassembly pathway. *PF22* knockdown in zebrafish causes a loss of dynein arms, cilia dysmotility, and a typical ciliopathy phenotype with axis curvature, pronephric cysts, hydrocephalus and situs inversus. We propose the existance of a conserved multi-step pathway for formation of assembly-competent dynein complexes, and that *PF22* (now renamed *DNAAF3*, “dynein axonemal assembly factor 3”) mutations cause PCD with situs inversus due to deficient cytoplasmic dynein assembly which in turn leads to missing outer arms in the axoneme.

